# Delivering malaria services during the COVID-19 pandemic: challenges and lessons from an international non-governmental organisation

**DOI:** 10.1093/inthealth/ihag004

**Published:** 2026-01-30

**Authors:** Elisabeth G Chestnutt, Madeleine Marasciulo, Godfrey Magumba, Kolawole Maxwell, Christian Rassi, Andrew Parkes, Charles Nelson, James K Tibenderana

**Affiliations:** Malaria Consortium, 244-254 Cambridge Heath Road, London E2 9DA, UK; Malaria Consortium, 8024 Upper Lake Dr, Raleigh, NC 27615, USA; Malaria Consortium, Plot 2A, Sturrock Road, Kololo, Kampala, Uganda; Malaria Consortium, 33 Pope John Paul II St, Maitama 904101, Abuja, Federal Capital Territory, Nigeria; Malaria Consortium, 244-254 Cambridge Heath Road, London E2 9DA, UK; Malaria Consortium, 244-254 Cambridge Heath Road, London E2 9DA, UK; Relief International, Haußmannstr. 198, 70188 Stuttgart, Germany; Malaria Consortium, 244-254 Cambridge Heath Road, London E2 9DA, UK; Malaria Consortium, 8024 Upper Lake Dr, Raleigh, NC 27615, USA; Malaria Consortium, 244-254 Cambridge Heath Road, London E2 9DA, UK

## Abstract

During the COVID-19 pandemic, our team implemented a coordinated, evidence-driven response to maintain malaria services. Key actions included establishing a dedicated taskforce, developing an outbreak management plan with four risk phases and adapting protocols to local contexts to enable safer delivery methods. In this article we present reflections from staff in decision-making roles during the COVID-19 pandemic. These experiences offer practical insights for other organisations preparing for future operational challenges.

## Introduction

On 30 January 2020, the World Health Organization (WHO) declared coronavirus disease 2019 (COVID-19) a public health emergency of international concern.^1^ Less than 2 months later it was recategorized as a pandemic.^2^ Researchers worked tirelessly to understand the novel RNA virus and its behaviour, trying to mitigate any impact. As public information increased, concerns grew about the impact of the disease in low-resource settings, including sub-Saharan Africa. The symptom similarities between COVID-19 and other prevalent diseases, coupled with struggling health systems, had the potential to create a perfect storm. In March 2020, the WHO urged countries to maintain malaria services, predicting disruptions could lead to a doubling of malaria deaths.^3^ During the pandemic, our team supported the delivery of malaria services in 11 countries across Africa and Asia. Here we share reflections from staff in decision-making roles at the time to highlight the challenges and enablers that supported the continuity of malaria services. Sharing these lessons can support organisations reflecting on their own preparedness for future operational challenges.

## Approach

We conducted an internal review of key operational documents and interviews with seven staff members who were part of organisational decision-making during the pandemic to capture lessons. Interviews were conducted in 2022 and lasted for approximately 1 h. Participants were asked about their initial actions and main priorities, data used to inform decisions, how policies differed across countries and the greatest challenges and enablers of success. Responses were grouped by theme and documentary evidence was used to contextualise and triangulate information including dates, actions and contents of guidance documents.

## Setting

In January 2020, as reports of a new infectious disease emerged, staff began tracking information about the disease to keep up to date. A COVID-19 taskforce was formally established with specific staff assigned to gather the most recent evidence and epidemiological statistics. At the time, data were scarce and underreporting was widespread. Consequently, in-country staff became a critical source of experiential information, helping to triangulate official global data sources. Driven by the mission to safely maintain services, the taskforce developed protocols and guidance to support country-level teams to safely continue operations.

## Actions

A global outbreak management plan was developed to guide decisions, with four risk phases—awareness, containment, protection and mitigation—each of which required a different response. To implement the outbreak management plan, the COVID-19 taskforce continued to collect data to enable evidence-based decision-making. Communication mechanisms were also set up to cascade information on restrictions or closures that could affect staff or program delivery. In collaboration with partners, country-specific guidance was developed and regularly updated and protocols were established to support continued service delivery while maintaining safety. Operating procedures were flexible to be responsive to new information and changing situations. Adaptations included delivering insecticide-treated nets and health education door to door instead of gathering households at health facilities and the recommendation that seasonal malaria chemoprevention (SMC) medicines be administered by caregivers rather than community distributors.^4^ The adaptations ensured the SMC program was implemented without major disruptions, supporting a planned expansion—covering 20 million children in 2021 compared with 6 million in 2019—which helped alleviate pressure on other health services.^4–6^ The adaptations also informed global guidance (e.g. the RBM Partnership to End Malaria’s guidance on SMC implementation during COVID-19).^7^

Alongside protocol development and program adaptation, country teams connected with Ministry of Health COVID-19 management and response units to advocate that malaria services be categorised as essential. This allowed staff to gain travel permits to continue activities during lockdowns. Teams also worked with governments to enable wider communication and strengthen community health systems. Community health workers (CHWs) were essential for continued service delivery when patients were unable to travel to health facilities. Recognising governments were prioritising hospitals and health facilities for personal protective equipment (PPE), we delivered masks, gloves, hand sanitisers and disinfectants at the community level to ensure CHWs and community members were protected.

Once developed, protocols and standard operating procedures were adapted to the local context, considering operational feasibility in remote and low-resource settings and coordination with national government approaches. While national guidance took precedence, where there was a conflict in approach, the more conservative way was followed.

## Lessons

### Enablers

Several factors facilitated the response.

#### Expertise

Several of the organisation’s staff were medically trained and had experience in pandemic preparedness, outbreak management and effective infection prevention and control practices. In addition, in 2018, the WHO released a checklist for pandemic influenza risk and impact management to support countries to develop emergency pandemic plans. In response, the organisation’s technical team had reviewed the checklist to identify how to support countries to assess their capabilities. At the same time, an organisational business continuity plan was developed that included regional epidemics as a risk to program delivery. This prior training and expertise enabled our team to critically evaluate information internally rather than waiting for external guidance to be published, resulting in faster decision-making and action. Furthermore, the organisation had experience implementing large, complex supply chains for SMC so staff were able to transfer this experience to source and deliver PPE and malaria supplies at a time when sourcing and shipping commodities were severely restricted.

#### Trust internally and externally

Internally, rapid decision-making was a product of trust that had been built within the organisation over many years. This trust allowed decisions to be made and signed off quickly without waiting for external guidance. Externally, the trusted relationships in place with ministries of health, national malaria programs and funders enabled the organisation to engage early and advocate positively to build a coordinated response. For example, the team in Nigeria was quick to purchase Zoom licences for the National Malaria Programme to maintain communication.

#### Cross-country learning

With staff based across different geographies, it was easier to capture information from a variety of sources and review it immediately. This enabled rapid triangulation of global information with local knowledge that ensured countries were correctly categorised and responses were appropriate.

#### Motivated local staff

The value of local staff was clear. Teams were able to remain at nearly full capacity and staff were highly motivated to support their countries and communities throughout the pandemic.

#### Funder flexibility

One key enabler was the flexible approach of funders during the pandemic, allowing budgets to be repurposed (e.g. reallocating travel budgets for purchasing PPE so services could be delivered safely) or advancing future funds for early use, which supported a rapid response.

Much of this flexibility was a result of trust in the organisation’s decision-making capabilities. Furthermore, unrestricted funding from individual donors increased during the pandemic, allowing the organisation to fill gaps quickly to maintain continuity of services.

### Challenges

Several key challenges also emerged:

#### Lack of data

With limited, incomplete and sometimes conflicting information there was significant uncertainty, meaning decisions were always judgement calls. Rapid diagnostic tests had to be developed and testing capacity scaled up. Data reporting lagged and decisions to deviate from normal operating practice had to be based on expertise and instinct, introducing risk.

#### Operating remotely

Travel restrictions posed a barrier to communication. New systems needed to be set up to enable staff to work remotely and communication infrastructure struggled with the surge in remote work. Over time, information sharing improved significantly. However, some aspects, including mentoring and training, remained challenging to deliver online. Although trainers could demonstrate practice, they were unable to observe participants to confirm understanding and quality. This was amplified by issues with connectivity, especially where there was insufficient bandwidth to keep cameras on.

#### Timing

The pandemic was declared approaching the beginning of the peak malaria transmission season in the Sahel. Protocols had to be adapted quickly to ensure time-sensitive programs, such as SMC, were delivered as planned to avoid reduced effectiveness. Additionally, PPE had to be procured and delivered at a time of exceptional global demand. Masks and hand sanitisers were often unavailable, expensive or of poor quality in a market flooded with counterfeit products and price gouging. Quality checks were needed to ensure products were high quality and culturally suitable, such as procuring alcohol-free hand sanitisers for communities that avoid alcohol for religious reasons.

#### Alignment of approach

Occasionally the protocols developed by the organisation took a more conservative approach to infection prevention and control than required locally (e.g. differing standards for masks and longer durations for hand washing) and negotiations with partners were needed.

## Conclusions

Since the downgrading of the COVID-19 crisis, the organisation has reviewed adaptations made during the pandemic to identify any that improved the efficiency or effectiveness of programs and systems. Although the pandemic phase has now passed, as a global health community we cannot be complacent. Epidemics happen. From our experience, building and maintaining strong trusted relationships and prioritising localisation are supportive when successfully navigating future challenges (Box 1). As a global community we must also strengthen surveillance capabilities, shore up health systems and build resilience within communities to reduce vulnerability to future emerging diseases.

Box 1. **Key lessons for practice**

- Invest in team cohesion: strong internal teams who already collaborate effectively are supportive in times of instability and uncertainty
- Build trusted partnerships: trusted relationships with governments, partners and funders are built over time not overnight
- Prioritise localisation: hiring local staff in country offices supports operational continuity and contextualised responses

## Data Availability

The data underlying this article will be shared upon reasonable request to the corresponding author.
